# Outpatient intravenous sedation for dental treatment in patients with special health care needs: a five-year retrospective study in Venezuela

**DOI:** 10.3389/froh.2026.1759205

**Published:** 2026-04-07

**Authors:** Mariana Morales-Chávez

**Affiliations:** Director of Dental Research Center, Dental School, Santa Maria University, Caracas, Venezuela

**Keywords:** autistic disorder, dental care for disabled, outpatients, sedation, intravenous, special care dentistry

## Abstract

**Background:**

Patients with special health care needs (SHCN) frequently present behavioral and medical conditions that complicate conventional dental treatment. This study aimed to describe the clinical profile, dental procedures, pharmacological protocols, and safety outcomes of outpatient intravenous sedation in SHCN patients treated in a specialized center in Venezuela.

**Materials and methods:**

A retrospective review was conducted of all SHCN patients who received outpatient intravenous sedation for dental treatment at a private clinic in Caracas, Venezuela, between January 2019 and December 2024. Demographic data, type of disability, American Society of Anesthesiologists (ASA) physical status, dental procedures performed, sedative and analgesic regimens, duration of treatment and sedation, number of sedation sessions, intraoperative oxygen saturation, and complications were extracted from electronic records. Only patients classified as ASA I–III with complete medical and dental charts were included. Data were analyzed using descriptive statistics (means and standard deviations for continuous variables; frequencies and percentages for categorical variables). Group comparisons, when performed, used chi-square or Fisher's exact tests for categorical variables and t tests or Mann–Whitney U tests for continuous variables, with a significance level of 0.05.

**Results:**

A total of 212 SHCN patients (70.8% male; mean age 11.1 ± 10.5 years) underwent 2,269 dental procedures under intravenous sedation. Autism spectrum disorder was the most frequent condition (34.0%), followed by dental phobia (16.5%), Down syndrome (16.0%), and very young uncooperative children (13.7%). Restorative procedures accounted for 52.2% of all treatments, preventive/periodontal procedures for 27.8%, and surgical interventions for 20.0%. The most common drug regimens were midazolam–fentanyl–ketamine (34.0%) and midazolam–fentanyl–propofol (27.4%). Oxygen saturation remained ≥90% in all but one transient episode, and minor complications occurred in 3.3% of sedations.

**Conclusions:**

Outpatient intravenous sedation was feasible for delivering comprehensive dental care to patients with special health care needs within a specialized outpatient clinic. A low observed complication rate and limited need for repeated sedation sessions were documented, supporting its use as a contextualized clinical approach when performed by trained teams under strict monitoring protocols.

## Introduction

Patients with special health care needs (SHCN) frequently present medical, developmental, or behavioral conditions that limit cooperation and increase the complexity of dental treatment. Behavioral management techniques can be effective for some of these patients; however, a substantial subgroup does not respond adequately and requires pharmacological approaches to enable safe and efficient dental care. Sedation has therefore become a key component of special care dentistry, where it is primarily used to control anxiety, pain, and excessive movement during procedures (1, 2).

Sedation is defined as a drug-induced depression of the central nervous system that reduces anxiety and facilitates dental treatment while preserving an adequate level of consciousness and protective reflexes (3). According to the American Academy of Pediatric Dentistry, the goals of sedation include promoting high-quality patient care, minimizing disruptive behaviors, enhancing the patient's overall experience, and ensuring that the child or adolescent returns to their pre-sedation physiological state (4). In 2018, multidisciplinary guidelines on moderate procedural sedation and analgesia emphasized the need for rigorous patient evaluation, continuous monitoring (including capnography when available), and the presence of trained personnel capable of recognizing and managing airway complications (5, 6).

Outpatient dental procedures under deep or moderate intravenous sedation offer several advantages over general anesthesia, including lower costs, shorter operating times, lower drug doses, and the ability to perform minimally invasive, conservative treatments in a dental office setting (7, 8). At the same time, sedation carries inherent risks, such as loss of airway patency, respiratory depression, cardiovascular instability, and adverse drug reactions, which may be more pronounced in pediatric and medically compromised populations (6, 7). Consequently, careful patient selection, appropriate pharmacological protocols, and strict adherence to monitoring standards are essential.

In this context, outpatient intravenous sedation has emerged as a relevant pharmacological strategy to facilitate dental treatment in patients with special health care needs, particularly in non-hospital settings. When appropriately indicated and performed under an anesthesiologist's supervision, intravenous sedation enables the delivery of comprehensive, minimally invasive dental care while avoiding the logistical complexity, higher costs, and limited availability associated with hospital-based general anesthesia. However, its use requires careful patient selection, standardized protocols, and strict monitoring, especially in medically compromised populations (9–12).

Despite the increasing use of intravenous sedation in dentistry, there are relatively few studies describing its application in patients with special health care needs in Latin America, particularly in office-based settings and under anesthesiologist-supervised protocols (8, 13–15) Existing reports often focus on specific disabilities, single drug regimens, or hospital-based services, limiting the generalizability of their findings to real-world outpatient dental practice (13–15). In this context, there is a need for region-specific data on the clinical profile of SHCN patients undergoing intravenous sedation, the types and duration of dental procedures performed, the pharmacological combinations used, and the incidence of complications.

Therefore, the objective of this study was to analyze the clinical records of patients with special health care needs treated under outpatient intravenous sedation in a specialized dental clinic in Venezuela, to (1) describe their demographic and medical characteristics, (2) characterize the types of disabilities and dental treatments performed, (3) summarize the intravenous sedative and analgesic regimens used, and (4) evaluate the safety and efficacy of outpatient intravenous sedation based on intraoperative oxygen saturation, complications, and the need for repeated sedations sessions.

## Materials and methods

### Study design and setting

This was a retrospective observational study (case series) including all patients with special health care needs who underwent outpatient intravenous sedation for dental treatment at a specialized dental clinic in Caracas, Venezuela, between January 2019 and December 2024.

### Ethical approval

The study protocol was reviewed and approved by the Research Ethics Committee of Santa María University, Caracas, Venezuela (CBFOUSM-300420253). Written informed consent was obtained from all adult patients, and from parents/guardians for minors and individuals with limited decision-making capacity.

### Participants and eligibility criteria

Patients were eligible if they met all of the following criteria:

Diagnosis of a medical, developmental, behavioral, or psychological condition that limited cooperation and required pharmacological management for dental treatment (patients with special health care needs). Indication for outpatient intravenous sedation for dental care. ASA physical status classification I, II, or III. Complete medical and dental records, including preoperative assessment, intraoperative monitoring, and postoperative notes.
Although outpatient intravenous sedation is most commonly indicated for ASA I–II patients, individuals classified as ASA III were included in this study only when their systemic condition was stable and well controlled, following a comprehensive preoperative evaluation by an anesthesiologist and an individualized risk–benefit assessment. In patients with special health care needs, behavioral, cognitive, and sensory challenges frequently preclude dental treatment under local anesthesia, making pharmacological management necessary.
In the context of a middle-income, developing country, access to hospital-based general anesthesia is often limited by availability and economic barriers, particularly for patients with disabilities who require comprehensive dental care. In selected, clinically stable ASA III patients, outpatient intravenous sedation was a feasible and accessible alternative to general anesthesia when the potential benefits outweighed the risks and strict monitoring and safety standards were met.
In the Venezuelan regulatory context, intravenous sedation outside the operating room is legally permitted provided it is performed under the direct supervision of a qualified anesthesiologist. All ASA III sedation procedures included in this study were conducted in compliance with this requirement, within a specialized dental clinic equipped to manage deep sedation, including continuous physiological monitoring, emergency preparedness, and a designated post-anesthesia recovery area.

Exclusion criteria were:

Patients classified as ASA IV or higher, as well as those requiring hospital-based general anesthesia, were excluded. Incomplete or missing key data in the clinical records.

### Variables and data collection

The following information was extracted from the records using a standardized data collection form:

Demographics: age (years), sex. Type of disability or condition: autism spectrum disorder (ASD), Down syndrome, cerebral palsy, intellectual disability, dental phobia, very young age/uncooperative child, cleft lip and palate, and other syndromic conditions. ASA physical status (I–III). Number of sedation sessions per patient. Dental procedures performed, categorized as: Restorative (resin restorations, sealants, pulpotomies, endodontic treatments, impressions for rehabilitation). Preventive and periodontal (fluoride applications, varnish, prophylaxis, scaling). Surgical (simple and complex extractions, frenectomies, minor soft-tissue surgery). Duration of dental treatment and sedation (minutes). Pharmacological regimens: type and combination of sedatives and analgesics (for example, midazolam alone; midazolam–propofol; midazolam–fentanyl; midazolam–fentanyl–ketamine; midazolam–fentanyl–propofol; midazolam–propofol–ketamine; midazolam–fentanyl–propofol–ketamine; ketamine–propofol). Intraoperative monitoring: peripheral oxygen saturation (SpO_2_), with documentation of any episodes <90%. Adverse events and complications: respiratory depression, desaturation, vomiting, paradoxical reactions, cardiovascular instability, infections (including septicemia), and any unplanned hospital referral. Need for new sedation sessions for completion of dental treatment.

### Sedation and monitoring protocol

All sedations were performed by an anesthesiologist trained in outpatient dental sedation, in collaboration with the dental team. Preoperative assessment included medical history, physical examination, ASA classification, and evaluation of airway and behavioral status. Patients fasted according to current guidelines for procedural sedation.

### Sedation protocol and pharmacological management

Outpatient intravenous sedation was administered and supervised by a qualified anesthesiologist, in collaboration with the dental team. Preoperative assessment included medical history, physical examination, ASA classification, and evaluation of airway and behavioral status. Patients fasted according to current guidelines for procedural sedation.

All sedative and analgesic doses were individualized based on patient weight, ASA physical status, medical condition, and the type and invasiveness of the dental procedure, using a dose–response approach rather than a fixed protocol.

The most commonly used agents included midazolam, fentanyl, ketamine, and propofol, either alone or in combination. Typical dosing ranges were as follows: midazolam (0.05–0.2 mg/kg), fentanyl (1 µg/kg), ketamine (0.5–1 mg/kg), and propofol (0.5–1.5 mg/kg). Continuous infusions were not routinely used. Sedation depth was achieved with an initial dose and maintained with intermittent intravenous boluses, adjusted according to patient response and procedural requirements.

For minimally invasive or non-painful procedures, lighter levels of sedation were intentionally maintained, whereas deeper levels of sedation were used for more invasive or painful interventions. When benzodiazepines, opioids, and ketamine were combined, this was performed selectively and based on individual patient characteristics. Ketamine was avoided in patients with a history of seizures or specific neurological disorders. In some cases, sedation was maintained with additional propofol boluses according to the complexity and duration of the dental treatment.

The depth of sedation was clinically titrated by the anesthesiologist using a dose–response approach based on patient behavior, physiological parameters, and procedural requirements. A formal sedation depth scale (such as the Ramsay Sedation Scale or the University of Michigan Sedation Scale) was not systematically recorded in the clinical records, as this study was retrospective and relied on routine documentation. Consequently, sedation depth was adjusted dynamically during each procedure to maintain adequate conditions for dental treatment while preserving cardiorespiratory stability.

Throughout all procedures, supplemental oxygen was provided via nasal cannula when indicated. Continuous monitoring included noninvasive blood pressure, pulse oximetry, heart rate, and respiratory rate.

At the end of the procedure, patients were observed in a recovery area until they met discharge criteria, including stable vital signs, adequate airway patency, and an appropriate level of consciousness. Caregivers received written postoperative instructions and an emergency contact number.

### Statistical analysis

Taking into account the nature of the variables, the measurement level at which the values of the variables are expressed, the way in which events occur, the number of groups or samples to be compared, and the sample size, the following tests were used for hypothesis testing, depending on whether or not the parametric assumptions were met: 1. The Kolmogorov–Smirnov and Shapiro–Will tests to verify the normality of the populations of origin of the samples in the case of quantitative variables. 2. The non-parametric Kruskal–Wallis test for the comparison of more than two independent samples; in cases of verification of the statistical significance of “difference in behavior between groups,” Dunn's test is used for pairwise comparisons between groups, adjusting the significance values using the Benferroni correction. 3. The Mann–Whitney *U* test for cases involving the comparison of two independent samples. As a decision rule in cases of null hypotheses (Ho), the *p*-value criterion associated with the contrast statistic was used, deciding to reject it in cases where the statistical significance was less than the set Significance Level (NS: *α* = 0.05), that is, in cases where the *p*-value is less than the significance level (*p* < 0.05) and a confidence level (CL) of 95% was used. The following software was used for processing, tabulation, and statistical calculations: Microsoft Office LTS Standard 2021 and IBM SPSS Statistics 27, in their Spanish versions.

## Results

### Study population

A total of 212 patients with special health care needs were included in the analysis. The mean age was 11.1 ± 10.5 years, with a median of 11 years and a range from 1 to 51 years. Most patients (71.7%) were younger than 12 years, followed by those aged 19–40 years (14.1%), 12–18 years (10.4%), and 41–51 years (3.8%). Males accounted for 70.8% (*n* = 150) and females for 29.2% (*n* = 62), and there was no statistically significant difference in age between sexes. The mean age for male patients was 10.0 ± 8.7 years, while for female patients it was 13.7 ± 13.8 years (Tables 1,2).

**Table 1:** Basic statistics of the age variable by sex.

Sex	Sample (*n*)	Mean $\left(\bar{x}\right)$	Median (Me)	S.D. (s)	CI 95%	Value		Range
						Higher (HV)	Lower (Lv)	(HV − Lv)
Total	212	11.1	7.0	10.5	(9.7; 12.5)	51	1	50
Male	150	10.0	7.0	8.7	(8.6; 11.4)	50	1	49
Femele	62	13.7	6.5	13.8	(10.2; 17.2)	51	1	50

Source: Own study.

**Table 2:** Distribution of patients by age group and sex.

Age group	Total (%)	Sex	
		Male (%)	Femele (%)
Total	212 100.0	150 (70.8)	62 (29.2)
Under 12	152 (71.7)	112 (73.7)	40 (26.3)
12–18	22 (10.4)	17 (77.3)	5 (22.7)
19–40	30 (14.1)	19 (63.3)	11 (36.7)
41–51	8 (3.8)	2 (25.0)	6 (75.0)

Source: Own study.

### Type of disability

The distribution of underlying conditions is shown in Table 3. Autism spectrum disorder (ASD) was the most prevalent diagnosis, observed in 72 patients (34.0%), followed by dental phobia in 35 patients (16.5%), Down syndrome in 34 patients (16.0%), and very young uncooperative children in 29 cases (13.7%). Cerebral palsy was present in 9 patients (4.2%), intellectual disability in 8 (3.8%), and cleft lip and palate in 7 (3.3%). An additional 18 patients (8.5%) had other rare syndromes, including Cri-du-Chat syndrome, partial trisomy of the short arm of the X chromosome, Rubinstein–Taybi syndrome, Norrie syndrome, Sotos syndrome, Cornelia de Lange syndrome, Lennox–Gastaut syndrome, and Moebius syndrome. (Table 3)

**Table 3:** Patients with special needs and types of conditions.

Condition	fi	%
Total	212	100.0
Autism	72	34.0
Down syndrome	34	16.0
Cerebral palsy	9	4.2
Intelectual disability	8	3.8
Phobia	35	16.5
Young age	29	13.7
Cleft lip palate	7	3.3
Other syndromes	18	8.5

### Number of sedation sessions

Most patients required only one sedation session to complete their dental treatment. Specifically, 52.7% of patients underwent a single sedation session, 28.8% required two sessions, and 12.9% required three sessions. A small proportion of patients required four (2.2%) or five sessions (2.7%). (Table 4)

**Table 4:** Number of sedation sessions per patient.

Number of Sedations	Frequency (fi)	Percentage (%)
Total	211	100.0
One	185	87.7
Two	19	9.0
Three	5	2.4
Four	1	0.5
Five	1	0.5

Significantly, each sedation session corresponded to a single dental visit during which all planned procedures for that visit were completed under one continuous intravenous sedation. Patients who underwent more than one sedation session did so at different times, often separated by months or years, because new or additional dental treatments became necessary over time, rather than because they underwent repeated sedations for the same treatment episode.

### Dental procedures and duration

A total of 2,269 dental procedures were performed under intravenous sedation. These were grouped into restorative, preventive/periodontal, and surgical treatments. Restorative procedures accounted for 1,397 interventions (52.2%), including resin restorations, sealants, pulpotomies, endodontic treatments, and impressions for dental rehabilitation, with a mean duration of 57 min. Preventive and periodontal procedures accounted for 437 treatments (27.8%), including fluoride applications, fluoride varnish, prophylaxis, and scaling, with an average duration of 40 min. The surgical group included 435 procedures (20.0%), mainly tooth extractions (*n* = 400; 92.0%), frenectomies (labial and lingual), removal of residual roots, and minor soft-tissue surgeries (e.g., gingivectomies). Minor surgical procedures lasted a mean of 36 min, whereas more complex surgeries required approximately 61 min (Table 5).

**Table 5:** Total treatments performed on patients during sedation, for type.

Type of Treatment	Total (%)	Number of sedations performed on patients				
		1st	2nd	3rd	4th	5th
Total	2.269 (100.0)	2,008	118	29	5	6
Restorers:	1.134 (50.0)	1,058	53	16	4	3
- Resins	804 (70.9)	761	32	11	—	—
- Ionomers	265 (23.4)	237	18	3	4	3
- Impressions	16 (1.4)	14	1	1	—	—
- Pulpectomies	42 (3.7)	40	1	1	—	—
- Root canals	7 (0.6)	6	1	—	—	—
Surgeries:	435 (19.2)	398	33	2	—	2
- Extractions	400 (92.0)	366	30	2	—	2
- Surgical eyelets	15 (3.4)	13	2	-	—	—
- Lingual frenectomy	5 (1.2)	5	—	—	—	—
- Labial frenectomy	4 (0.9)	4	—	—	—	—
- Root residues	8 (1.8)	7	1	—	—	—
- Gingivectomy	2 (0.5)	2	—	—	—	—
- Periodontal Surgery	1 (0.2)	1	—	—	—	—
Prevention:	700 (30.8)	655	32	11	1	1
- Sealants	263 (37.6)	246	11	6	—	—
- Tartretomy	161 (23.0)	141	15	3	1	1
- Prophylaxis	119 (17.0)	114	4	1	—	—
- Fluorine	92 (13.1)	91	1	—	—	—
- Fluorine varnish	45 (6.4)	44	—	1	—	—
- Chlorhexidine	7 (1.0)	7	—	—	—	—
- Root Scraping	13 (1.9)	12	1	—	—	—

Source: Own Study.

A single 22-year-old male patient underwent five sedations in the corresponding columns.

### Dental procedures, gender, age, and type of disability

When performing the comparative analysis, it was determined that when contrasting the medians of the number of restorative treatments performed on patients according to gender, age, and type of disability using the Mann–Whitney *U* test and Kruskal–Wallis test, the *p*-values were *p* = 0.745, *p* = 0.107, and *p* = 0.616, respectively, indicating that there was no statistically significant relationship.

On the other hand, when comparing the medians of the number of surgical treatments performed on patients according to gender, age, and medical condition using the Mann–Whitney *U* test and Kruskal–Wallis test, the *p*-values were *p* = 0.302, *p* = 0.861, and *p* = 0.010, respectively, indicating that there was no statistically significant relationship for gender and age. Still, there was a type of disability.

When comparing the medians of the number of preventive treatments performed on patients by gender using the Mann–Whitney *U* test, the *p*-value was 0.249, indicating no statistically significant difference.

About the median number of preventive procedures by age, no calculations are made since 81.99% of patients are in the 1–18 age group.

Finally, when evaluating the medians of preventive procedures by disability type, the *p*-value was 0.001, indicating that the significance level (NS = 0.05) is significant. Based on this result, we rejected the null hypothesis of no difference. We concluded that there is statistical evidence of a significant difference between groups, with the medians of the number of preventive procedures differing by the diagnosed pathology. As the test was significant, multiple comparisons were performed pairwise using Dunn's test (the most recommended and versatile method, which also adjusts for multiple comparisons using the Bonferroni correction). In our case, once the procedure was performed, statistical significance was found between the groups (*p* < 0.05), these groups being the following (Tables 6–8):

1. Dental phobia–Autism: *p* = 0.018
2. Dental phobia–Other syndromes: *p* = 0.044
3. Dental phobia–Age-related caries: *p* = 0.006
4. Dental phobia–Down syndrome: *p* = 0.001.

**Table 6:** Restorative treatments performed during sedations by Age groups and Sex.

Restorative Procedure	Total (%)	Under 12 (%)	12–18 (%)	19–40 (%)	41–51 (%)
GENERAL TOTAL	1,392 (100.0)	1,009 (72.5)	145 (10.4)	209 (15.0)	29 (2.1)
Resin (Composite)	771 (55.4)	576 (74.7)	74 (9.6)	108 (14.0)	13 (1.7)
Glass Ionomer	265 (19.0)	195 (73.6)	26 (9.8)	32 (12.1)	12 (4.5)
Sealant	263 (18.9)	155 (58.9)	41 (15.6)	63 (24.0)	4 (1.5)
Pulpectomy	42 (3.0)	42 (100.0)	—	—	—
Reconstruction	13 (0.9)	11 (84.6)	—	2 (15.4)	—
Endodontics	7 (0.5)	1 (14.2)	3 (42.9)	3 (42.9)	—
Impressions	16 (1.2)	14 (87.5)	1 (6.2)	1 (6.2)	—
Male Patients	Total (%)	Under 12 (%)	12–18 (%)	19–40 (%)	41–51 (%)
TOTAL MASCULINE	1,019 (73.2)	756 (74.2)	125 (12.3)	138 (13.5)	—
Resin (Composite)	555 (54.5)	428 (77.1)	58 (10.5)	69 (12.4)	—
Glass Ionomer	202 (19.8)	157 (77.7)	24 (11.9)	21 (10.4)	—
Sealant	198 (19.4)	115 (58.1)	38 (19.2)	45 (22.7)	—
Pulpectomy	33 (3.2)	33 (100.0)	—	—	—
Restoration/Recon.	10 (1.0)	8 (80.0)	1 (10.0)	1 (10.0)	—
Endodontics	5 (0.5)	1 (20.0)	3 (60.0)	1 (20.0)	—
Impressions	13 (1.3)	11 (84.6)	1 (7.7)	1 (7.7)	—
Female Patients	Total (%)	Under 12 (%)	12–18 (%)	19–40 (%)	41–51 (%)
TOTAL FEMININE	373 (26.8)	253 (67.8)	20 (5.4)	71 (19.0)	29 (7.8)
Resin (Composite)	216 (57.9)	148 (68.5)	16 (7.4)	39 (18.1)	13 (6.0)
Glass Ionomer	56 (15.0)	38 (67.9)	2 (3.6)	4 (7.1)	12 (21.4)
Sealant	54 (14.5)	36 (66.7)	3 (5.6)	11 (20.4)	4 (7.4)
Pulpectomy	9 (2.4)	9 (100.0)	—	—	—
Reconstruction	5 (1.3)	4 (80.0)	—	1 (20.0)	—
Endodontics	2 (0.5)	—	—	2 (100.0)	—
Impressions	3 (0.8)	3 (100.0)	—	—	—

**Table 7:** Surgical procedures performed during sedation by Age groups and Sex.

Surgical Procedure	Total (%)	Under 12 (%)	12–18 (%)	19–40 (%)	41–51 (%)
GENERAL TOTAL	422 (100.0)	279 (66.3)	33 (7.9)	82 (19.1)	28 (6.7)
Exodontia	400 (95.0)	269 (67.6)	31 (7.8)	81 (19.8)	19 (4.8)
Root Remnants	11 (2.6)	1 (9.1)	1 (9.1)	9 (81.8)	—
Lingual Frenectomy	5 (1.2)	5 (100.0)	—	—	—
Labial Frenectomy	4 (1.0)	3 (75.0)	1 (25.0)	—	—
Gingivectomy	2 (0.2)	2 (100.0)	—	—	—
Male Patients	Total (%)	Under 12 (%)	12–18 (%)	19–40 (%)	41–51 (%)
TOTAL MALE	291 (69.0)	208 (71.5)	24 (8.2)	53 (18.2)	6 (2.1)
Exodontia	275 (94.5)	200 (72.7)	22 (8.0)	48 (17.5)	5 (1.8)
Root Remnants	6 (2.1)	—	1 (16.7)	5 (83.3)	—
Lingual Frenectomy	5 (1.7)	5 (100.0)	—	—	—
Labial Frenectomy	3 (1.0)	2 (66.7)	1 (33.3)	—	—
Gingivectomy	2 (0.7)	1 (50.0)	—	—	1 (50.0)
Female Patients	Total (%)	Under 12 (%)	12–18 (%)	19–40 (%)	41–51 (%)
TOTAL FEMALE	131 (31.0)	71 (54.2)	9 (6.9)	29 (22.1)	22 (16.8)
Exodontia	125 (95.4)	69 (55.2)	9 (7.2)	33 (26.4)	14 (11.2)
Root Remnants	5 (3.8)	1 (20.0)	—	4 (80.0)	—
Labial Frenectomy	1 (0.8)	1 (100.0)	—	—	—

**Table 8:** Preventive treatments performed on patients during sedations by age groups and sex.

Preventive Procedure	Total (%)	Under 12 (%)	12–18 (%)	19–40 (%)	41–51 (%)
GENERAL TOTAL	343 (100.0)	222 (65.9)	46 (12.8)	52 (13.9)	23 (7.4)
Cleaning	62 (16.9)	36 (58.0)	6 (9.7)	15 (24.2)	5 (8.1)
Prophylaxis	67 (18.3)	56 (83.5)	6 (9.0)	4 (6.0)	1 (1.5)
Scaling (Tartrectomy)	49 (13.4)	16 (32.7)	12 (24.5)	16 (32.6)	5 (10.2)
Fluoride	48 (13.1)	37 (77.1)	7 (14.6)	3 (6.2)	1 (2.1)
Fluoride Varnish	25 (7.4)	22 (81.5)	2 (7.4)	—	1 (7.4)
Cleaning + Chlorhexidine	4 (1.1)	—	1 (25.0)	1 (25.0)	2 (50.0)
Cleaning + Fluoride Varnish	10 (2.7)	9 (90.0)	—	—	1 (10.0)
Cleaning + Fluoride	6 (1.6)	4 (66.6)	1 (16.7)	1 (16.7)	—
Scaling + Prophylaxis + Fluoride	13 (3.5)	7 (53.8)	3 (23.1)	2 (15.4)	1 (7.7)
Scaling + Chlorhexidine	3 (0.8)	—	—	2 (66.7)	1 (33.3)
Scaling + Fluoride Varnish	2 (0.5)	—	2 (100.0)	—	—
Scaling + Prophylaxis	6 (1.7)	3 (50.0)	1 (16.7)	2 (33.3)	-
Scaling + Root Planing	6 (1.7)	—	2 (33.3)	2 (33.3)	2 (33.4)
Root Planing	7 (3.1)	—	2 (28.6)	3 (42.8)	2 (28.6)
Prophylaxis + Fluoride	25 (7.1)	24 (96.0)	1 (4.0)	—	—
Prophylaxis + Fluoride Varnish	8 (2.2)	8 (100.0)	—	—	—
Male Patients	Total (%)	Under 12 (%)	12–18 (%)	19–40 (%)	41–51 (%)
TOTAL MASCULINE	247 (72.0)	172 (70.5)	37 (14.1)	33 (13.2)	5 (2.2)
Cleaning	47 (20.3)	28 (59.6)	6 (12.7)	11 (23.4)	2 (4.3)
Prophylaxis	52 (22.9)	43 (82.7)	5 (9.6)	4 (7.7)	-
Scaling (Tartrectomy)	34 (15.0)	14 (41.2)	10 (29.4)	9 (26.5)	1 (2.9)
Fluoride	35 (16.3)	28 (80.0)	6 (17.1)	1 (2.9)	—
Fluoride Varnish	17 (7.5)	16 (94.1)	1 (5.9)	—	—
Cleaning + Fluoride Varnish	9 (3.6)	9 (100.0)	—	—	—
Cleaning + Fluoride	5 (2.0)	4 (80.0)	—	1 (20.0)	—
Scaling + Proph. + Fluoride	10 (4.1)	7 (70.0)	2 (20.0)	1 (10.0)	—
Scaling + Prophylaxis	4 (1.6)	3 (75.0)	—	1 (25.0)	—
Scaling + Root Planing	2 (0.8)	—	1 (50.0)	1 (50.0)	—
Root Planing	2 (0.8)	—	1 (50.0)	1 (50.0)	—
Prophylaxis + Fluoride	19 (7.7)	18 (94.7)	1 (5.3)	—	—
Prophylaxis + Fluoride Varnish	8 (3.3)	8 (100.0)	—	—	—
Female Patients	Total (%)	Under 12 (%)	12–18 (%)	19–40 (%)	41–51 (%)
TOTAL FEMININE	96 (28.0)	50 (58.3)	9 (5.0)	19 (20.0)	18 (16.7)
Cleaning	15 (15.6)	8 (53.3)	—	4 (26.7)	3 (20.0)
Prophylaxis	15 (15.6)	13 (86.7)	1 (6.7)	—	1 (6.7)
Scaling (Tartrectomy)	15 (15.6)	2 (13.3)	2 (13.3)	7 (46.7)	4 (26.7)
Fluoride	13 (13.5)	9 (69.2)	1 (7.7)	2 (15.4)	1 (7.7)
Fluoride Varnish	8 (8.3)	6 (75.0)	1 (12.5)	—	1 (12.5)
Cleaning + Chlorhexidine	4 (4.2)	—	1 (25.0)	1 (25.0)	2 (50.0)
Scaling + Proph. + Fluoride	3 (3.1)	—	1 (33.3)	1 (33.3)	1 (33.4)
Scaling + Root Planing	4 (4.2)	—	1 (25.0)	1 (25.0)	2 (50.0)
Root Planing	5 (5.2)	—	1 (20.0)	2 (40.0)	2 (40.0)
Prophylaxis + Fluoride	6 (6.3)	6 (100.0)	—	—	—

### ASA classification and intraoperative safety

According to the American Society of Anesthesiologists (ASA) classification, most patients were classified as ASA II, indicating mild systemic conditions compatible with outpatient sedation. ASA I patients represented a smaller proportion, and only a minority were classified as ASA III, with controlled but significant systemic diseases. Outpatient intravenous sedation was not provided to ASA IV patients in this setting. Throughout all procedures, peripheral oxygen saturation remained stable; no patient experienced sustained desaturation below 90%, and only one transient episode of SpO_2_ < 90% was documented and resolved without sequelae, supporting the protocol's overall safety.

### Sedative medications and drug combinations

A total of 212 sedation procedures were analyzed with respect to pharmacological regimens (Table 6). The most frequent combinations were midazolam–fentanyl–ketamine, used in 72 cases (34.0%), and midazolam–fentanyl–propofol, used in 58 cases (27.4%). Other regimens included midazolam–fentanyl–propofol–ketamine (39 cases; 18.4%), midazolam–propofol (17 cases; 8.0%), midazolam–fentanyl (16 cases; 7.5%), midazolam alone (7 cases; 3.3%), midazolam–propofol–ketamine (2 cases; 0.9%), and ketamine–propofol (1 case; 0.5%). Together, the two most common combinations accounted for more than 60% of all sedation procedures, indicating a preference for balanced regimens based on benzodiazepines combined with short-acting anesthetic and analgesic agents (Table 9).

**Table 9:** Medication and drug combinations.

Drug combinations	fi	%
Total	212	100.0
- Midazolam	7	3.3
- Midazolam y Propofol	17	8.0
- Midazolam y Fentanyl	16	7.5
- Midazolam, Fentanyl y Ketamine	72	34.0
- Midazolam, Propofol y Ketamine	2	0.9
- Midazolam, Fentanyl y Propofol	58	27.4
- Midazolam, Fentanyl, Propofol y Ketamine	39	18.4
Ketamine y propofol	1	0.5

Source: Own study.

### Complications and paradoxical reactions

The overall incidence of complications was low. Only one patient presented a paradoxical reaction to midazolam. Minor adverse events included vomiting in approximately 2% of patients, transient respiratory depression in 0.66%, and one case of septicemia (0.66%) occurring after periodontal treatment despite antibiotic prophylaxis. All complications were managed successfully, with no need for unplanned hospital admission or long-term sequelae.

## Discussion

### Overview of main findings

This study provides a comprehensive description of outpatient intravenous sedation for dental treatment in patients with special health care needs (SHCN) in a specialized center in Venezuela. The majority of patients were children and adolescents, predominantly male, with autism spectrum disorder (ASD), dental phobia, and Down syndrome as the most frequent indications for sedation. Restorative procedures accounted for more than half of all treatments, followed by preventive/periodontal and surgical interventions, which reflects the predominance of minimally invasive and conservative approaches in this setting. The overall incidence of complications was low, with only minor adverse events and one transient episode of desaturation, supporting the safety and feasibility of intravenous sedation when performed by trained teams under strict monitoring (6–8, 15–18).

### Safety, ASA classification, and complications

The distribution of ASA physical status in this series, with most patients classified as ASA II and a smaller proportion as ASA I and III, indicates appropriate patient selection for outpatient intravenous sedation. This profile is consistent with recommendations that prioritize ASA I–II patients and carefully selected ASA III patients to minimize respiratory and cardiovascular risks (15–18) The fact that no sustained desaturation below 90% was observed and that only one transient episode of hypoxemia was rapidly resolved without sequelae suggests that the monitoring and emergency response protocols were effective. These results align with previous reports of low complication rates in intellectual disability and pediatric populations undergoing dental intravenous sedation when managed by experienced teams under standardized guidelines (6, 7, 15, 16, 18).

The incidence of complications observed in this study (approximately 3.3%, including vomiting, transient respiratory depression, and one case of septicemia) is comparable to or lower than rates reported in similar cohorts (16–18). Minor respiratory events are among the most frequently described adverse effects in pediatric and SHCN sedation, often associated with opioids and deep levels of sedation, underscoring the importance of careful titration and continuous monitoring (6, 7, 15, 16). The occurrence of septicemia after periodontal treatment, despite antibiotic prophylaxis, highlights the need for strict medical–dental evaluation and reinforced preventive strategies in patients at risk of bacteremia (17, 18).

### Profile of disabilities and clinical implications

In this series, ASD was the leading indication for intravenous sedation, followed by dental phobia, Down syndrome, and very young, uncooperative children. This distribution is consistent with the literature, where autism and severe anxiety are among the main reasons for using sedation in dental care for SHCN patients (8, 14, 16, 19) The high proportion of ASD patients reflects the substantial behavioral and sensory challenges that frequently preclude conventional behavior management techniques and necessitate pharmacological support (13, 16, 19).

Patients with Down syndrome constituted a significant subgroup and often presented cardiovascular comorbidities, in agreement with previous studies reporting high rates of congenital and acquired heart disease in this population (14, 17). This finding reinforces the need for systematic preoperative cardiovascular assessment, careful ASA classification, and, when indicated, antibiotic prophylaxis to reduce the risk of infective complications (14, 17, 20). The inclusion of very young children who were unable to cooperate, as well as patients with rare syndromes, highlights the role of intravenous sedation as a flexible tool that can be adapted to heterogeneous clinical and behavioral profiles in special care dentistry (7, 8, 13, 14).

The heterogeneity of the special health care needs population included in this study represents both a clinical reality and a methodological challenge. Patients with autism spectrum disorder, dental phobia, Down syndrome, very young uncooperative children, and other syndromic conditions differ substantially in behavioral profiles, medical comorbidities, sensory processing, and anesthetic risk. Consequently, variations in sedation requirements, procedural complexity, and perioperative management are expected across diagnostic categories. In the present study, the inclusion of a broad spectrum of conditions reflects real-world outpatient dental practice in a specialized center, where individualized sedation protocols are tailored to patient-specific needs rather than standardized by diagnosis. Similar approaches have been reported in previous retrospective studies in special care dentistry, which have prioritized descriptive analyses of clinical practice over subgroup comparisons (3, 4, 8).

### Sedation regimens and pharmacological considerations

The most frequently used drug combinations in this study were midazolam–fentanyl–ketamine and midazolam–fentanyl–propofol, which together accounted for more than 60% of all sedations. These regimens reflect a balanced approach, combining benzodiazepines for anxiolysis and amnesia, opioids for analgesia, and either propofol or ketamine for hypnosis or dissociative anesthesia (13, 16, 19, 21). The use of such multimodal combinations allows dose reduction of individual agents and may contribute to rapid recovery and lower rates of adverse events when administered by trained professionals (13, 16, 19, 21).

Previous studies have shown that children and patients with neurodevelopmental disorders, particularly ASD, may require higher doses of propofol or other sedatives to achieve adequate depth of anesthesia, possibly due to altered neurobehavioral and pharmacodynamic profiles (16, 19, 21). The present findings, with frequent use of balanced regimens and low complication rates, support the feasibility of tailoring sedation protocols according to the patient's diagnosis, medical status, and anticipated treatment duration. However, they also underscore the importance of vigilant monitoring and readiness to manage respiratory depression, especially when combining opioids with propofol (6, 7, 15, 16, 19, 21).

### Dental procedures, treatment duration, and outpatient feasibility

The high proportion of restorative procedures (over half of all treatments) and the substantial number of preventive/periodontal interventions highlight that intravenous sedation was used not only for urgent surgical needs but also to deliver comprehensive, minimally invasive dental care (7, 13, 15). The longer mean duration of restorative treatments compared with preventive and minor surgical procedures supports the decision to opt for intravenous sedation rather than general anesthesia, as it allows complete treatment in a single session for many patients while maintaining a favorable safety profile (7, 13, 15).

The finding that more than half of patients completed their treatment in a single sedation session, while only a small proportion required four or five sessions, suggests that individualized planning and prioritization of procedures were effective. This outcome is clinically relevant because each additional sedation increases costs, logistical demands, and potential cumulative risk, particularly in medically compromised populations (8, 15, 20). These results, in combination with the low rate of complications, support the role of outpatient intravenous sedation as a viable alternative to hospital-based general anesthesia for SHCN patients when infrastructure, personnel, and monitoring standards are adequate (7, 8, 15–18).

### Behavioral impact and need for repeated sedation

An essential aspect of sedation in pediatric and SHCN dentistry is its potential long-term impact on behavior and dental anxiety. Benzodiazepines such as midazolam produce anterograde amnesia, reducing the likelihood that patients will retain traumatic memories of dental treatment and thereby interrupting the cycle of fear and avoidance (21–23). The relatively low proportion of patients who required multiple sedations in this study may reflect, at least in part, the combination of positive, anxiety-free experiences under sedation with systematic preventive strategies and caregiver education (8, 20, 23).

Recent evidence suggests that structured preventive programs and regular follow-up can reduce the need for new sedation sessions by controlling caries risk and periodontal disease progression, particularly in vulnerable groups (20, 23). The present results are consistent with this concept and support an integrated approach in which sedation is not used in isolation, but rather as part of a broader model that includes oral hygiene reinforcement, dietary counseling, and tailored recall intervals for SHCN patients (7, 8, 13, 20, 23).

### Limitations and future directions

This study has several limitations that should be acknowledged. First, its retrospective design relies on the completeness and accuracy of clinical records, which may introduce information bias and limit the analysis of some variables, such as exact drug dosages, depth of sedation, or subtle behavioral outcomes. Second, this is a single-center study conducted in a specialized clinic, which may limit the generalizability of the findings to other settings with different resources, sedation protocols, or patient populations. Third, the study is primarily descriptive. At the same time, some statistical comparisons were performed; however, it was not designed to identify predictors of complications or the need for repeated sedation using robust multivariate models (6–8, 15–18, 20).

Future research should include multicenter, prospective studies comparing different sedation regimens, monitoring strategies, and preventive care models in SHCN populations. Additionally, standardized measures of dental anxiety, quality of life, and behavioral adaptation could provide deeper insight into the long-term impact of sedation beyond immediate procedural outcomes. Finally, economic evaluations comparing outpatient intravenous sedation with hospital-based general anesthesia would help clarify cost-effectiveness and inform health policy decisions in resource-limited settings (7, 13, 15, 20).

An additional limitation of this study is the absence of a standardized sedation depth score in the clinical records, which precluded a formal classification of sedation levels as moderate or deep. As a result, the analysis focused on observed clinical outcomes and safety parameters rather than on comparative assessments of sedation depth (10, 12, 16).

## Conclusion

This retrospective case series describes the use of outpatient intravenous sedation in a specialized dental clinic to deliver comprehensive restorative, preventive, and surgical dental care to patients with special health care needs. The study population consisted predominantly of children and adolescents with autism spectrum disorder, dental phobia, and Down syndrome, reflecting the clinical reality of patients who frequently present significant behavioral, cognitive, or medical barriers to conventional dental treatment.

Within this single-center setting, a low observed incidence of complications and only minor adverse events were documented when outpatient intravenous sedation was performed following strict preoperative assessment, continuous intraoperative monitoring, and direct supervision by an anesthesiologist. No sustained episodes of oxygen desaturation or unplanned hospital admissions were recorded, supporting the feasibility of this approach under controlled clinical conditions.

Most patients completed their planned dental treatment within a single sedation session. At the same time, only a small proportion required multiple sedations over time, generally due to the development of new treatment needs rather than incomplete care during a single visit. These observations suggest that, in selected patients and within appropriately equipped centers, outpatient intravenous sedation may be a feasible option for facilitating access to dental care, without implying superiority or equivalence relative to hospital-based general anesthesia or other anesthetic modalities.

The findings of this study should be interpreted in light of its retrospective design, single-center setting, and the heterogeneity of the population with special health care needs included. Although outpatient intravenous sedation can play an essential role in the dental management of selected patients, the choice between sedation and general anesthesia must be individualized, taking into account medical risk, treatment complexity, resource availability, and ethical considerations.

Further prospective, multicenter, and comparative studies are warranted to define better the safety profile, optimal indications, and relative roles of outpatient intravenous sedation and general anesthesia across different subgroups of patients with special health care needs.

## Data Availability

The raw data supporting the conclusions of this article will be made available by the authors, without undue reservation.
